# How to Be Acceptable

**Published:** 1875-07

**Authors:** 


### HOW TO BE ACCEPTABLE.

If we could only impress upon all mankind the fact that a sacred duty which devolves upon each individual is to keep himself or herself pure, sweet and acceptable to those about them at all times, we should feel that we had accomplished a work of priceless value. Of course we cannot do this, nor can we expect to influence any large proportion of the people, in the direction of that cleanliness which so nearly approximates godliness. But we do stimulate a select few to greater care of themselves, to greater consideration for the tastes and feelings of others, and in this we have a sweet and lasting reward. Thousands of our young readers will, by and by, reflect that to our teachings they owe something of their good manners, not a little of their good morals, and very much of their good habits, and they will, some way, thank us for our earnestness in their behalf. So we remember that when we preach temperance, and cleanliness, and a life of thoughtful purity, we are teaching our readers an all important lesson, and one which cannot be too early learned.

There is a great deal of selfishness in the world, and this trait is manifested in nothing more than in personal habits. It ought, for example, to be a sufficient inducement to any man to abandon the use of tobacco, to know that the habit makes him offensive to all the world. To the right minded man it surely would, for, disguise it as you will, the habit of using tobacco by eating it, or burning it in a pipe or in a roll, or putting it in the nose, *does* make the person so employing it, very dirty, very offensive in smell, and very disagreeable. The offence does not cease for days after the poison is abandoned. Its odor is wonderfully noxious and wonderfully lasting. It is simply detestable. Those persons who contaminate themselves with this dangerous and unclean herb, ought to be placed in a colony by themselves, without permission to approach cleanly human beings. By this means, and by confining marriages among tobacco users strictly to the members of the colony, the poisoned race would speedly die out, because strong and healthy offspring are not possible from such a union. Disease, imbecility and deformity of body and mind and conscience—these are the fruits garnered in the offspring of parents addicted to this selfish vice.

In thus teaching our friends how to be acceptable, we are inculcating a still higher moral—the moral of a pure life. For impurity in thought or act or word is always an offense, always objectionable. We demand of all, the young as well as well as the old that they preserve themselves pure, blameless and acceptable, and free from the contaminations which beset humanity everywhere.

---

An able man shows his spirit by gentle words and resolute actions; he is neither hot nor timid.

---

Friendship is the medicine for all misfortune, but ingratitude dries up the fountain of all goodness.

